# Paracrine Mechanisms Involved in the Control of Early Stages of Mammalian Spermatogenesis

**DOI:** 10.3389/fendo.2013.00181

**Published:** 2013-11-26

**Authors:** Pellegrino Rossi, Susanna Dolci

**Affiliations:** ^1^Dipartimento di Biomedicina e Prevenzione, Università degli Studi di Roma Tor Vergata, Rome, Italy

**Keywords:** primordial germ cells, spermatogonial stem cells, spermatogenesis, meiosis, growth factors, paracrine control, signal transduction, gene expression

## Abstract

Within the testis, Sertoli-cell is the primary target of pituitary FSH. Several growth factors have been described to be produced specifically by Sertoli cells and modulate male germ cell development through paracrine mechanisms. Some have been shown to act directly on spermatogonia such as GDNF, which acts on self-renewal of spermatogonial stem cells (SSCs) while inhibiting their differentiation; BMP4, which has both a proliferative and differentiative effect on these cells, and KIT ligand (KL), which stimulates the KIT tyrosine-kinase receptor expressed by differentiating spermatogonia (but not by SSCs). KL not only controls the proliferative cycles of KIT-positive spermatogonia, but it also stimulates the expression of genes that are specific of the early phases of meiosis, whereas the expression of typical spermatogonial markers is down-regulated. On the contrary, FGF9 acts as a meiotic inhibiting substance both in fetal gonocytes and in post-natal spermatogonia through the induction of the RNA-binding protein NANOS2. Vitamin A, which is metabolized to Retinoic Acid in Sertoli cells, controls both SSCs differentiation through KIT induction and NANOS2 inhibition, and meiotic entry of differentiating spermatogonia through STRA8 upregulation.

## Brief Introduction: Paracrine Control of Fetal Male Germ Cell Development

The control of the germ cell fate by paracrine factors secreted by the surrounding somatic environment already starts in the fetal life in the period of germ cell specification, independently from the influence of the hypothalamic-pituitary axis. Bone Morphogenetic Protein 4 (BMP4) has been shown to induce primordial germ cell (PGC) formation, to act as a PGC survival and localization factor within the allantois (1) and as a mitogen in *in vitro* cultured PGCs (2). During PGC specification in the extraembryonic mesoderm, SOX2 induction is required for the transcriptional regulation of KIT expression in PGCs (3). KIT is a tyrosine-kinase receptor, which is activated by KIT Ligand (KL), a growth factor expressed by the surrounding somatic environment. KL/KIT interaction is essential in the fetal period both during the specification of PGCs and for their proliferation and migration [(3–7), and references therein]. KIT expression is then down-regulated both in fetal oocytes undergoing meiosis and in gonocytes, which stop to proliferate after germ cell sex determination. Sertoli cells can prevent meiotic entry of gonocytes through the production of paracrine factors acting as meiotic inhibiting substances. The best characterized meiotic inhibiting substance produced by fetal Sertoli cells is Fibroblast Growth factor 9 (FGF9). FGF9 is a SRY/SOX9-dependent growth factor crucial for male sex differentiation acting on the somatic compartment of the fetal testis (8, 9). However, FGF9 also acts directly on male fetal gonocytes by upregulating levels of the RNA-binding protein NANOS2 (10, 11). NANOS2 prevents meiosis through the post-transcriptional regulation of key genes involved in the meiotic program (10, 12, 13). Recently, it has been shown that the meiosis-preventing activity of FGF9 in the fetal testis is mediated, at least in part, by NODAL, a member of the TGF-β family, and its partner Cripto (14–16).

In the same period in which FGF9 is expressed during testis determination, Sertoli cells produce an enzyme, CYP26B1, which degrades Retinoic Acid (RA) of mesonephric origin, in order to block Stimulated by Retinoic Acid 8 (STRA8) expression, and, as a consequence, to prevent premature gonocyte entry into meiosis (17–20). Although the identification of RA as the CYP26B1 substrate in the fetal testis (required for STRA8 induction and meiosis initiation in the fetal ovary) has been questioned (21), most of the available data in the literature support the role of RA as a master inducer of the mitotic-meiotic switch in germ cells (22). In line with this evidence is the finding that RA treatment down-regulates NANOS2 expression in fetal gonocytes (10).

## Paracrine Control of Post-Natal Male Germ Cell Development

**Figure 1:**
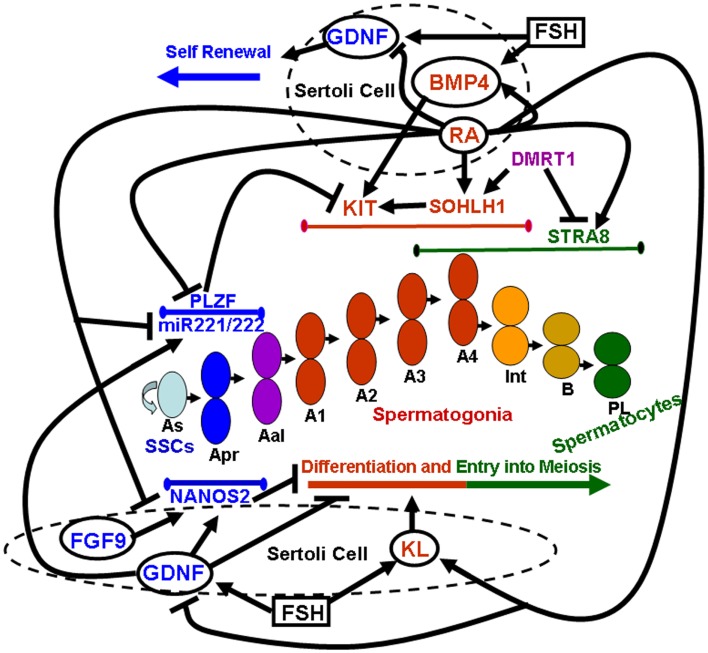
**Sertoli-cell controlled paracrine mechanisms acting on the early stages of mammalian spermatogenesis**. Paracrine factors secreted by Sertoli cells (whose membrane is represented by dashed circles) are enclosed within solid line circles. Follicle stimulating hormone (FSH) is enclosed within a solid line square. Endogenous factors expressed by germ cells are represented by non-enclosed words. Blue colors refer to paracrine and endogenous factors that promote self-renewal of spermatogonial stem cells (SSCs) and inhibit spermatogonial differentiation and/or meiotic entry. Red colors refer to paracrine and endogenous factors that promote spermatogonial differentiation. Purple colors refer to endogenous factors which promote spermatogonial differentiation but at the same time inhibit meiotic entry. Green colors refer to endogenous factors that drive entry into meiosis. Lines delimited by small ellipsoids refer to the stage of expression of the germ cell endogenous factors involved in either self-renewal of SSCs and inhibition of differentiation (blue colors) or in differentiation (red colors) and meiotic entry (green colors). The succession of the various types of germ cells during the earliest stages of mouse spermatogenesis is represented in the center of the image: As, a single spermatogonia; Apr, a paired spermatogonia; Aal, a aligned spermatogonia; A1, A2, A3, A4, type A1–A4 spermatogonia; Int, intermediate spermatogonia; B, type B spermatogonia; PL, pre-leptotene spermatocytes.

Pituitary gonadotropins, FSH, and LH, were originally identified for their essential role in ovarian function, as the stimulator of follicular activity and the inducer of follicular luteinization, respectively (23). Later on, it became clear that the same hormones play important roles also in testicular function, FSH being involved in the induction of spermatogenesis at puberty, and LH being the main inducer of androgen production (24). Spermatogenesis is a highly ordered differentiative process that occurs under FSH and androgen control. Sertoli cells, the only known targets for these hormones in the seminiferous tubules, mediate hormone action on spermatogenesis by controlling the germinal stem cell niche and by creating a suitable environment for the complex developmental events of germ cell proliferation and differentiation. Sertoli cells directly orchestrate these complex events through both membrane intercellular communications and the production of growth factors and cytokines that act directly on the germ cell compartment. In the following paragraphs we will focus on the better characterized Sertoli-cell controlled paracrine mechanisms acting on the early stages of mammalian spermatogenesis, which are schematically summarized in Figure 1.

### Maintenance of the germ stem cell niche

Spermatogonial stem cells (SSCs) are the direct descendants of fetal gonocytes. In the testis, SSCs are a subpopulation of undifferentiated spermatogonia residing in the basal layer of the seminiferous epithelium. Their mitotic expansion allows continuous production of germ cells committed to differentiation. One of the specific properties of SSCs and other undifferentiated spermatogonia that distinguishes them from differentiating spermatogonia is the expression of the Glial cell line-derived neurotrophic factor (GDNF)-family receptor α1 (GFRα1) and the c-Ret receptor tyrosine-kinase, which are both required for signaling in response to the Sertoli-cell-derived GDNF (25–28). GDNF has been shown to be essential for fate determination of SSCs, since in aging males heterozygotes for GDNF deletion, testes appear devoided of germ cells and show a phenotype similar to Sertoli-cell-only syndrome (25). Furthermore, overexpression of GDNF in mouse testes appeared to stimulate self-renewal of stem cells and block spermatogonial differentiation, inducing a seminomatous phenotype (25, 27). GDNF-induced activation of AKT and MEK signaling pathways in SSCs leads to increased generation of reactive oxygen species (ROS) generated by NAPDH oxidase 1, and apparently (contrary to their alleged detrimental role for spermatogenesis) ROS stimulate proliferation and self-renewal of SSCs through the activation of p38 and JNK MAPKs (29). Thus, GDNF is important for SSCs self-renewal, and, at the same time negatively controls their differentiation. This notion has been recently challenged by the finding that GFRα1-positive chained spermatogonia (A paired and A aligned) are more numerous than GFRα1-positive A single spermatogonia, which are thought to represent the major SSCs reservoir in the mouse testis (30). However, GDNF signaling is essential to maintain NANOS2 expression in SSCs, and it has been proposed that this RNA-binding protein, besides its well-established role in preventing meiosis in fetal gonocytes, is also important to prevent spermatogonial differentiation in the post-natal testis (31). Overall, it is clear that GDNF mainly acts in positively regulating the proliferation of SSCs and maintenance of their undifferentiated state. Importantly, FSH and its second messenger cyclic AMP (cAMP) have been reported to stimulate GDNF expression in Sertoli cells (32, 33), which is instead down-regulated by RA treatment (33). These evidences suggest that that GDNF might be one of the paracrine factors that influences SSCs proliferation and population size under the control of the hypothalamic-pituitary axis.

### Control of spermatogonial differentiation

Undifferentiated SSCs (A single spermatogonia) have been described as single cells that are able both to renew themselves and to produce more differentiated A paired spermatogonia. The A paired cells then divide into A aligned spermatogonia that further differentiate into A1 spermatogonia (34). Appearance of A1 (differentiating) spermatogonia coincides with regain of the expression of KIT, encoding the receptor for KL (35–38). KIT mediates proliferation, survival, and differentiation in type A spermatogonia (33, 39–41). Upon KIT expression, spermatogonia become sensitive to KL produced by Sertoli cells (39, 42) and undergo a definite number of proliferative cycles, forming the A2-A4, intermediate, and B spermatogonia, before entering meiosis. The temporal appearance of KIT expression and of KL sensitivity in mouse spermatogonia, between 4 and 7 days postpartum (dpp) (33, 35, 36, 40), marks the switch from the A aligned spermatogonia to the A1-B differentiating cell types. Indeed, KIT is universally considered the most important marker that distinguishes differentiating spermatogonia from their undifferentiated precursors, including SSCs. Thus, paracrine factors in the testicular environment that stimulate KIT expression in mitotic germ cells play an essential role for the start of spermatogenesis at puberty. One of the paracrine signals involved in this event is BMP4, which is produced by Sertoli cells very early in the post-natal life, and whose expression is positively regulated by cAMP and RA (33, 43). Its receptor ALK3 and the SMAD5 transducer are expressed in undifferentiated spermatogonia, and *in vitro* treatment of these cells with BMP4 exerts both mitogenic and differentiative effects, inducing [^3^H]thymidine incorporation and KIT expression both at the RNA and protein levels (43). As a result of the latter event, KIT-negative spermatogonia acquire sensitivity to KL (43). Since SSCs are able to renew themselves and at the same time to progress through differentiation (i.e., to the KIT-dependent stages of proliferation), BMP4 could be one of the factors that regulates such process. Alternatively, BMP4 could act on a subset of undifferentiated spermatogonia that have lost SSC features, i.e., that have entered the differentiative stage but are not yet KIT-positive. In agreement with the first possibility, BMP4 addition, on the opposite of GDNF, was shown to impair *in vitro* maintenance of mouse primary SSCs (44). Moreover, more recently BMP4 was shown to induce differentiation and KIT expression in a rat SSC cell line (45). In the adult testis, BMP4 has been reported to be produced by spermatogonia, but not by Sertoli cells (46), suggesting that it might work as a paracrine-autocrine factor modulating the establishment of the cycle of the seminiferous epithelium.

Another well-established paracrine factor involved in spermatogonial differentiation is the Vitamin A derivative RA. Mice kept on a diet deficient on vitamin A (VAD mice) or lacking vitamin A derivatives are sterile because the seminiferous tubules contain only undifferentiated KIT-negative spermatogonia, indicating a role of vitamin A in spermatogonia differentiation (38, 47). RA functions inside the nucleus recognizing two different classes of retinoid receptors. Both classes (RARs and RXRs) consist of three types of receptors, α, β, and γ, encoded by distinct genes and transduce RA signal by binding directly to RA-responsive elements. During post-natal development, each RAR is detected predominantly in a specific cell type of the seminiferous epithelium: RARα in Sertoli cells, RARβ in round spermatids and RARγ in type A spermatogonia (48). RARα conditional ablation in Sertoli cells showed germ cell apoptosis and seminiferous epithelium dysfunctions related to the disruption of Sertoli cells cyclical gene expression, which preceded testis degeneration (49). It has been reported that during the first, prepubertal, spermatogenic cycle RALDH-dependent synthesis of RA by Sertoli cells is indispensable to initiate differentiation of A aligned into A1 spermatogonia, and that this effect is mainly mediated by autocrine action of RA through RARα in the somatic compartment (50). However, RA (either the all-trans or the 9-cis Retinoic isomers) treatment *in vitro* exerts a direct effect on the differentiation of mitotic germ cell compartment by promoting KIT expression in undifferentiated spermatogonia (33, 51). This effect has been confirmed *in vivo* by the observation that targeted ablation of RARγ impairs the A aligned to A 1 transition in the course of some of the seminiferous epithelium cycles (52). Altogether these data indicate that RA favors spermatogonial differentiation through a direct action on spermatogonia and an indirect action mediated by changes in the expression pattern of paracrine factors such as KL, BMP4, and GDNF secreted by Sertoli cells (33).

Due to its importance for promoting expansion of differentiating spermatogonia, KIT expression in SSCs is subjected to a very tight transcriptional control. Promyelocytic Leukemia Zinc Finger (PLZF, also known as ZFP145, or ZBTB16) is a DNA sequence-specific transcriptional repressor that can exert local and long-range chromatin remodeling activity through the recruitment of DNA histone deacetylases and through the action of several nuclear corepressors (53). PLZF is specifically expressed in SSCs, and male PLZF knock-out (KO) mice show progressive spermatogonia depletion due to the deregulated expression of genes controlling the switch between self-renewal and differentiation (54–56). PLZF represses both endogenous KIT expression and expression of a reporter gene under the control of KIT regulatory elements (57). A discrete sequence of the KIT promoter, required for PLZF-mediated KIT transcriptional repression, was demonstrated to be bound by PLZF *in vitro* and also *in vivo*, by using chromatin immunoprecipitation (ChIP) of spermatogonia. Moreover, a 3-bp mutation in this PLZF binding site abolishes the responsiveness of the KIT promoter to PLZF repression In agreement with these findings, a significant increase in KIT expression was found in the undifferentiated spermatogonia isolated from PLZF KO mice (57). Thus, one mechanism by which PLZF maintains the pool of SSCs is through a direct repression of KIT transcription, thus acting as a gatekeeper of spermatogonial differentiation. RA was shown to trigger downregulation of PLZF in SSCs (58), which might be part of the mechanisms which triggers up-regulation of KIT during spermatogonial differentiation.

Positive regulators of KIT transcription in spermatogonia are two b-Helix-Loop-Helix (HLH) transcription factors specifically expressed in germ cells, SOHLH1 (Spermatogenesis and Oogenesis HLH1), and SOHLH2. Both SOHLHs have been involved in the differentiation of spermatogonia and oocytes (59–64). In the male, deletion of each transcription factor leads to the disappearance of KIT-expressing spermatogonia in the prepuberal testis. An expression study of SOHLH1 and SOHLH2 during fetal and post-natal development showed a strong positive correlation between KIT and the two transcription factors in post-natal spermatogonia (65). SOHLH2 was found enriched mainly in undifferentiated spermatogonia, whereas SOHLH1 expression was maximal in KIT-dependent stages. Reporter gene expression driven by sequences contained within the KIT promoter and first intron was strongly up-regulated in transfection experiments overexpressing either SOHLH1 or SOHLH2, and co-transfection of both factors showed a cooperative effect (65). *In vivo*, co-immunoprecipitation results evidenced that the two proteins interact and overexpression of both factors increased endogenous KIT expression. Using ChIP analysis, SOHLH1 was found to occupy discrete bHLH binding site containing regions within the KIT promoter in spermatogonia chromatin (64, 65). Interestingly, expression of SOHLH1 was increased in post-natal mitotic germ cells by treatment with All-trans RA (65), which might be another mechanisms through which vitamin A derivatives triggers KIT up-regulation and spermatogonial differentiation. Using conditional gene targeting, it has been shown that loss of the Doublesex-related transcription factor DMRT1 in spermatogonia causes a precocious exit from the spermatogonial program and entry into meiosis (66). Apparently, DMRT1 acts in differentiating spermatogonia by restricting RA responsiveness, directly repressing transcription of the meiotic inducer STRA8, and activating transcription of SOHLH1, thereby preventing meiosis and promoting spermatogonial development (66). In agreement with the direct role played by SOHLH1 in regulating KIT transcription (65), a drastic reduction of KIT expression in spermatogonia was evident in testes from DMRT1 conditional KO mice (66).

Retinoic acid can up-regulate KIT expression in spermatogonia also at the post-transcriptional level, by interfering with the action of two X-linked microRNAs, miR-221 and miR-222 (67). Since miR-221/222 negatively regulate both KIT mRNA and KIT protein abundance in spermatogonia, impaired expression of these microRNAs in mouse undifferentiated spermatogonia induces transition from a KIT-negative to a KIT-positive state and loss of stem cell capacity to regenerate spermatogenesis. Undifferentiated spermatogonia overexpressing miR-221/222 were found to be resistant to RA-induced transition to a KIT-positive state and incapable of differentiation *in vivo* (67). Moreover, growth factors that promote maintenance of undifferentiated spermatogonia, such as GDNF, were found to up-regulate miR-221/222 expression. On the contrary, exposure to RA down-regulates miR-221/222 abundance (67). In conclusion, RA promotes progression of SSCs to differentiating spermatogonia through different mechanisms, all of which positively influence KIT expression: downregulation of PLZF and of miR-221/222, and up-regulation of SOHLH1.

### Control of spermatogonial expansion

KIT ligand/KIT interaction is essential during post-natal stages of spermatogenesis for the expansion of the differentiating spermatogonia pool. KL, expressed by Sertoli cells, stimulates proliferation of differentiating type A1-A4 spermatogonia both by inducing their progression into the mitotic cell cycle and by reducing their apoptotic rate. This effect is exerted by the activated KIT tyrosine-kinase using as signal transducers both PI3K-AKT and MEK-ERK1/2 (39, 40, 68). The role of KIT/KL in the maintenance and proliferation of differentiating spermatogonia has been highlighted by a mouse genetic model with a point mutation of KIT that eliminates the PI3K docking site (Y719F) through a single bp change (69, 70). While PGC specification and proliferation in both sexes is not compromised during embryonic development, KIT(Y719F)/KIT(Y719F) males are sterile due to the lack of spermatogonia proliferation during the prepuberal period and an arrest of spermatogenesis at the pre-meiotic stages. The KIT/KL system is also an important mediator of the influence of hypothalamic-pituitary axis on the spermatogenic process. Indeed, the expression of the mRNA for KL is induced by FSH in prepuberal mouse Sertoli cells cultured *in vitro*, through an increase in cAMP levels (39, 42). The cAMP-dependent increase in KL expression in Sertoli cells is mainly due to direct activation of transcription from proximal promoter elements within the KL gene (71). Stage-dependent induction of KL mRNA expression by FSH has also been observed in the adult rat testis (72), and the maximal levels of KL mRNA induction are observed in stages of the seminiferous epithelium which show the maximal sensitivity to FSH stimulation, and in which type A spermatogonia are actively dividing. Interestingly, the soluble and membrane forms of KL, produced by alternative splicing, are differentially expressed during testis development. Sertoli cells from prepuberal mice mainly express the mRNA encoding for the transmembrane form, while the mRNA encoding for the soluble form is expressed at higher levels later, in coincidence with the beginning of the spermatogenic process, and the two transcripts are expressed at equivalent levels in the adult testis (39). Moreover, FSH and/or cAMP analogs, beside increasing KL mRNA levels, also modify the splicing pattern of the two isoforms in cultured mouse Sertoli cells in favor of the mRNA encoding for the soluble form (39). In agreement with these observations is the finding that the highest levels of the transmembrane form of KL are detected immunohistochemically in stages VII-VIII of the mouse seminiferous epithelium (73), which are the less sensitive to FSH stimulation in the adult testis (74). It has been hypothesized that the transmembrane form of KL could be physiologically relevant for the progression through the blood-testis barrier of mitotic germ cells entering the first meiotic prophase at stages VII-VIII (5). Moreover, even though at the onset of meiosis KIT expression in male germ cells ceases at both the RNA and protein levels (5), KL/KIT interaction, besides its well-established role in the expansion of differentiating type A spermatogonia, is also important for entry into the meiotic program, i.e., the transition from type B spermatogonia to pre-leptotene spermatocytes, as discussed in the next paragraph.

### Control of entry into meiosis

Retinoic acid acts in a bimodal mode to promote the spermatogenic process. Indeed, besides its important role in promoting progression of SSCs to differentiating spermatogonia through activation of KIT expression, RA also promotes expression of the meiotic inducer STRA8 in spermatogonia (33, 51). Besides RA of Sertoli-cell origin, it has been reported that also RA synthesized by pre-meiotic spermatocytes cell autonomously induces meiotic initiation through controlling the RAR-dependent expression of STRA8 in the same cells (50). Targeted ablation of STRA8 revealed a crucial role for this gene in the initial stages of the meiotic process in post-natal male germ cells, either in the transition from type B spermatogonia/pre-leptotene to leptotene spermatocytes (75), or in slightly later stages of the meiotic prophase, with mutant leptotene spermatocytes undergoing a premature mitotic-like chromosome condensation (76). The mechanisms through which STRA8 regulates the initial stages of meiosis in both sexes are currently unknown. However, the role played by STRA8 in male meiosis appears to be different from that played in the induction of the meiotic process in the fetal ovary, in which STRA8 ablation leads to an arrest of pre-meiotic DNA synthesis in pre-leptotene oocytes (18), whereas the last round of germ cell DNA synthesis appears not be affected in STRA8-deficient pre-leptotene spermatocytes (75, 76). RA was found to increase meiotic entry of mouse KIT-positive differentiating spermatogonia *in vitro*, as evaluated by both morphological and biochemical criteria (33). Increased expression of STRA8 and of early meiotic markers, such as DMC1, accompanied the morphological switch from spermatogonia to pre-leptotene and leptotene spermatocytes. RA treatment also increased STRA8 expression in *in vitro* cultured KIT-negative undifferentiated spermatogonia, which included SSCs, but this was not followed by induction of meiotic entry, suggesting that spermatogonial competence to enter meiosis is acquired only during the differentiative stages in which they undergo KIT-dependent mitotic divisions (33). Transcriptome analysis of *in vitro* cultured differentiating spermatogonia stimulated with recombinant KL revealed a pattern of RNA expression compatible with the qualitative changes of the cell cycle that occur during the subsequent cell divisions in type A and B spermatogonia, i.e., the progressive lengthening of the S phase and the shortening of the G2/M transition (41). Moreover, KL treatment was found to up-regulate in differentiating spermatogonia the expression of early meiotic genes, and to down-regulate at the same time typical spermatogonial markers, suggesting an important role for KL/KIT interaction in the transition from the mitotic to the meiotic cell cycle, and also an active role in the induction of meiotic differentiation (41). Indeed, morphological and biochemical analysis of *in vitro* cultured spermatogonia treated with KL revealed an induction of STRA8 and DMC1 expression and of meiotic entry, evaluated as a dramatic increase in the number of pre-leptotene and leptotene spermatocytes similar to the one induced by RA treatment (33). The effect of RA and KL on meiotic entry did not appear to be additive, implying that these factors converge on common signal transduction pathways to exert this effect. Indeed, similarly to KL, RA treatment induced KIT autophosphorylation, MEK-ERK1/2 and PI3K-AKT activation, and selective inhibitors of any of these pathways inhibited the biochemical and morphological signs of meiotic entry. Thus, together with genomic effects leading to increased expression of KIT in spermatogonia and of KL in Sertoli cells, RA also exerts rapid non-genomic effects in differentiating spermatogonia and converge with KL on common KIT-dependent signaling pathways for the induction of meiotic entry (33).

In order to ensure the homeostasis of the spermatogenic process, paracrine mechanisms, and endogenous effectors which negatively regulate spermatogonial differentiation and the onset of the meiotic process in post-natal spermatogenesis must coexist with positive inducers such as RA and KL. One of these paracrine mechanisms is analogous to the one operating to prevent meiosis onset in the fetal testis, and involves FGF9 expression in the somatic environment of the seminiferous epithelium and expression of the RNA-binding protein NANOS2 in pre-meiotic germ cells. In the post-natal testis, NANOS2 was found to be specifically expressed at both the RNA and protein level in KIT-negative undifferentiated spermatogonia, but not in KIT-positive differentiating spermatogonia, nor in meiotic or postmeiotic germ cells (10). FGF9 stimulation of *in vitro* cultured differentiating spermatogonia resulted in a dramatic induction of NANOS2 expression and inhibition of the morphological and biochemical signs of entry into meiosis, without apparent effects on the expression of STRA8, whereas RA treatment resulted in a deep inhibition in the levels of NANOS2 expression in undifferentiated spermatogonia, together with the previously described stimulation of STRA8 expression (10). Thus, together with playing an essential role in preventing meiosis of gonocytes in the male fetal testis, FGF9 acts as an inhibitor of meiotic differentiation through the upregulation of NANOS2 also in post-natal male mitotic germ cells.

## Future Perspectives

Obviously there must be also a paracrine influence of germ cells on Sertoli-cell production of factors involved in the local control of spermatogenesis, but, up to now, little information is available in the literature about these germ cell-generated signals. On the other hand, Sertoli cells are clearly the only mediators of the influence of the hypothalamic-pituitary axis on the spermatogenic process. FSH drives both Sertoli-cell secretion of GDNF, on one side, and of BMP4 and KL, on the other side. This actually fits with the double role exerted by the pituitary hormones, as inducers of spermatogenesis at puberty (through the local mediation of BMP4 and KL), but at the same time as essential for its maintenance and quantitative output (through GDNF stimulation of SSCs self-renewal). The factors which locally control the balance between GDNF vs. BMP4 and KL secretion by Sertoli cells in response to FSH might be germ cell-generated signals, and they must be the object of further studies.

Another puzzling observation is that FGF9 exerts opposite effects in KIT-positive differentiating spermatogonia with respect to those elicited by RA and KL signaling. Indeed, it is intriguing to notice that KL and FGF9 act on the same germ cell type stimulating receptor tyrosine-kinase activities (and thus presumably partially shared signal transduction pathways), yet they exert opposite effects (differentiation and promotion of meiosis vs. prevention of meiotic entry). It will be very important to dissect the differences in intracellular signaling elicited in differentiating spermatogonia by these two antagonistic growth factors and the downstream cascade of events that lead to RA/KL-mediated induction of meiotic entry and FGF9-mediated inhibition of the same process. For instance, it will be interesting to characterize the subtypes of FGF receptors expressed in spermatogonia, and to investigate whether activation of NODAL signaling is involved in FGF9 action in post-natal male germ cells as it has been reported for male fetal gonocytes (14–16). Preliminary results from our laboratory indicate that both FGF9 and KL stimulate transient ERK1/2 activation in spermatogonia, but PI3K-dependent AKT activation is elicited by KL, but not by FGF9 (V. Tassinari, P. Rossi, and S. Dolci, unpublished results). This might be of particular importance, in light of the notion that in the mouse testis, as mentioned previously, a point mutation of KIT that eliminates the PI3K docking site cause a total block of the spermatogenic process between 8 and 10 dpp (69, 70), coinciding with of the onset of meiosis in the male germ cell line, and that PI3K inhibitors completely block induction of meiotic entry elicited *in vitro* by RA and/or KL treatment of differentiating spermatogonia (33).

## Conflict of Interest Statement

The authors declare that the research was conducted in the absence of any commercial or financial relationships that could be construed as a potential conflict of interest.
